# Bleeding During Veno-Venous ECMO: Prevention and Treatment

**DOI:** 10.3389/fmed.2022.879579

**Published:** 2022-05-23

**Authors:** Johannes Kalbhenn, Barbara Zieger

**Affiliations:** ^1^Department of Anesthesiology and Critical Care, Faculty of Medicine, Medical Center—University of Freiburg, Freiburg im Breisgau, Germany; ^2^Division of Pediatric Hematology and Oncology, Department of Pediatrics and Adolescent Medicine, Faculty of Medicine, Medical Center—University of Freiburg, Freiburg im Breisgau, Germany

**Keywords:** acquired von Willebrand syndrome, ARDS, ECMO, bleeding, thrombosis, prevention, treatment

## Abstract

Veno-venous extracorporeal membrane oxygenation (vvECMO) has become a routine treatment for severe lung failure in specialized centers. Spontaneous bleeding complications, however, are observed in 30–60% of patients during vvECMO treatment. Bleeding increases mortality by factors 2–3. Anticoagulation in combination with several acquired bleeding disorders caused by the mechanical pump and the foreign layer of the extracorporeal system contribute to the risk of bleeding. In this review, the mechanisms of the underlying pathologies and the route from diagnosis to treatment are described.

## Introduction and History

Several intra- and extra pulmonary triggers can cause pulmonary inflammation leading to lung failure and consecutive acute respiratory distress (1). Today, the different etiologies and the severity are summarized as acute respiratory distress syndrome (ARDS) (2). When a severe form of ARDS develops, mechanical lung-protective (3) ventilation alone may not sufficiently supply patients with oxygen or adequately support them to eliminate carbon dioxide. Increasing pressure or oxygen concentration at the ventilator will probably aggravate the problem: the more invasive ventilator settings are adjusted, the more damage will be caused to the lungs (ventilator-induced lung injury, VILI) (4). To overcome this vicious circle, Hill and Bramson presented the first system for bedside extracorporeal carbon dioxide removal, the Bramson membrane lung in 1972 (5). The Bramson membrane lung was a huge machine connected to the patient in a veno-arterial approach to support him for 3 days. During this period, 10 units of packed red blood cells, 8 units of frozen plasma, and 13 units of platelet concentrates had to be transfused. In the 1970s, the idea of extracorporeal lung support was evaluated in clinical trials (6); however, it took several years to establish lung support as veno-venous rather than veno-arterial procedure (7). In the following years, “extracorporeal membrane oxygenation” (ECMO) was applied only in several specialized centers worldwide, because the invasive procedure to implant the cannulas, the thromboembolic complications, and the short half-life of the components prevented this procedure from becoming routine. The coincidence of two different events around the year 2010 paved the way for the renaissance of extracorporeal lung support: The worldwide influenza A H1N1 pandemic causing severe respiratory failure in thousands of patients (8) and the discovery of polymethylpentene (9) as ideal material to produce cheap, effective, and long-lasting fibers for oxygenator membranes (10). Veno-venous ECMO (vvECMO) provided a survival benefit for well-selected patients with severe ARDS during the H1N1 pandemic (8, 11). In a meta-analysis including two prospective randomized trials performed before the SARS-CoV2-pandemic, 90-day mortality was significantly lowered by vvECMO compared with conventional management in patients with severe ARDS (12).

Modern vvECMO systems drain venous blood from the inferior or superior cava vein or the right atrium by negative pressure, accelerate the blood in a centrifugal pump, and press the blood with positive pressure along a membrane made of hollow fibers in which gas exchange takes place (13–15). After oxygenation and decarboxylation, the blood is returned into the right atrium, right ventricle, or even in the pulmonary artery, defined by the type of cannulas used (10, 16–19). The same systems but with different cannulation sites may be applied in veno-arterial ECMO, used for heart, and lung support (20). As several other issues complicate perfusion, turbulences, and coagulation in veno-arterial systems leading to various additional aspects, this article exclusively focuses on coagulation disorders during vvECMO.

According to findings from Kalbhenn, Zieger et al. typical clinical bleeding at vvECMO can be attributed primarily to severe disorders of primary hemostasis (21–23). First described between 2014 and 2018 in intensive care patients at Freiburg University Medical Center, acquired von Willebrand syndrome (AVWS), thrombocytopenia, and platelet dysfunction are characteristic findings that can be detected in virtually all vvECMO patients. Secondary waste coagulopathy with a deficiency of fibrinogen and factor XIII becomes apparent. The blood flow through a vvECMO system generates negative pressures of up to minus 200 mmHg in the supplying tube system. A positive pressure is then generated in the pump which can be up to plus 200 mmHg depending on the tube diameter, hematocrit, and pump speed. In the pump, but also when flowing through oxygenators or by pressing the recirculated blood through the cannula which narrows for technical reasons, considerable turbulence and tensions are generated that affect the blood components. Shear forces unfold the von Willebrand factor (vWF) high-molecular-weight (HMW) multimers and thus present the A2 domain at which a specific metalloprotease [A disintegrin and metalloproteinase with a thrombospondin type 1 motif, member 13 (ADAMTS13)] attaches (24). The physiologic function of ADAMTS13 is the degradation of unfolded HMW vWF multimers. Excessive cleavage into vWF which is missing the high-molecular-weight (HMW) multimers and which shows a far less hemostatic activity occurs. The increased degradation leading to a significant reduction or complete loss of HMW vWF multimers is typical for “acquired von Willebrand syndrome” (AVWS). AVWS is a bleeding disorder similar to von Willebrand disease that occurs when there are deficiencies in VWF concentration, structure, or function as a result of acquired conditions (25). It is characterized by deficiency or complete loss of HMW vWF multimers increasing the risk of spontaneous bleeding from mucous membranes and, for example, catheter insertion sites (21, 26, 27). Routine coagulation analyses (e.g., international normalized ratio or activated partial thromboplastin time) are unsuited in detecting AVWS. The diagnosis of AVWS requires documentation of reduced vWF binding to either collagen (vWF collagen binding capacity, vWF: CB) or to platelet glycoprotein Ib receptors (vWF activity, vWF:A) in relation to vWF antigen (vWF: Ag). Test kits for quick determination of vWF: Ag and vWF: A meanwhile are commercially available and may routinely be provided by every clinical laboratory. In case of decreased vWF: CB/vWF: Ag or vWF: A/vWF: Ag ratio during vvECMO, AVWS is the most likely pathology. To confirm this diagnosis and for differentiation between AVWS and some types of inherited von Willebrand disease, however, multimer analysis by sodium dodecyl sulfate-agarose (SDS) gel electrophoresis is required (28, 29). SDS gel electrophoresis can only be performed in specialized coagulation laboratories and is relatively time-consuming.

The above mentioned pressure phenomena not only induce AVWS but also have impact on the relatively fragile platelet membrane inducing thrombocytolysis (“accelerated platelet destruction”) with consecutive thrombocytopenia (30–32). The mechanical destruction of the blood cells leads to the detachment of small membrane particles from platelets and also erythrocytes. These microparticles can present thrombogenic antigens and activate plasmatic coagulation (33, 34). Consecutively, this leads to the activation and accumulation of more platelets. It is not surprising that the higher the pump flow is set at the ECMO, the more pronounced is the platelet consumption (35, 36). Also contributing to thrombocytopenia is platelet consumption due to the activation of the platelets on the foreign materials of the tubing and oxygenators (57).

Besides the determination of platelet count, also platelet function was determined (37) in a subgroup of vvECMO patients (21). Basic platelet function test aggregometry was performed after stimulation of platelets with ristocetin, collagen, adenosine diphosphate (ADP), and epinephrine (38). Regardless of which of these substrates, a relevant hypoaggregation resulted which was still detectable days after ECMO explantation (21). This functional test was complemented by flow cytometry (39) to investigate the expression of several platelet receptors and secretion of α- and δ-platelet granules (40, 41) after staining of receptors with fluorescein-labeled monoclonal antibodies. In addition, vWF-binding capacity was determined using fluorescein-labeled monoclonal antibodies against vWF and fibrinogen. These investigations revealed highly reduced CD62 and CD63 expression (hinting to impaired α- and δ-granule secretion, respectively) and a reduced vWF-binding capacity (21). Taken together, these findings represent a reduced activatability of platelets which may be due to mechanical damage by ECMO or by exhaustion due to former activation of the platelets in the cannula or in the ECMO.

## Anticoagulation and ECMO

Because of the increased risk of thrombosis within the extracorporeal system leading to malfunction and embolism, therapeutic anticoagulation was a paradigm not questioned during vvECMO for a long time (42). This was based on experiences from cardiac bypass surgery when patients without therapeutic anticoagulation had a larger waste of fibrinogen and platelet concentrates compared to those who were anticoagulated (43). Anticoagulation is established with unfractionated heparin (UFH) in the majority of patients (44). Modern cannulas, pumps, and membranes are coated with heparin or phosphorylcholine (45). A phosphorylcholine coating is considered to mimic the biological endothelial surface because phosphorylcholine is an integral component of the cell membrane (46). Phosphorylcholine coating (PHISIO) of tubes, cannula, oxygenator, and filter is associated with statistically unchanged vWF: ristocetin cofactor activity during cardiopulmonary bypass (47). Without doubt, the risk of bleeding when using anticoagulants is increased and has to be carefully weighed against the risk of thrombosis. Gail Annich focused on this dilemma in 2015 (48). The safe and effective use of low-molecular-weight heparin (LMWH) like enoxaparin and minimized anticoagulation concepts meanwhile have been proven in several studies (49, 50). As heparin-induced thrombocytopenia (HIT), a potential life-threatening reaction after recurrent exposition to heparin (51) may occur, and some centers use direct thrombin inhibitors like bivalirudin or argatroban instead of UFH or LMWH for anticoagulation. Independent of the type of anticoagulant, monitoring of anticoagulation still is sophisticated as acquired coagulation disorders may not be assessed by or influence routine laboratory markers (see below). Panigada et al. quoted that “ECMO […] does not change coagulation parameters” but “major bleedings occurred” in an observational study in 2016 (61). It is obvious that bleeding caused by ECMO was probably induced by factors not observed by routine coagulation tests such as prothrombin time or aPTT.

## Bleeding During ECMO

Hemorrhagic diathesis has been described in nearly every ECMO patient. Registry data report bleeding complications in 22% (52) up to 45% (53) of vvECMO patients. Cannulation site and surgical site bleeding are the most frequent bleeding localizations, followed by pulmonary and spontaneous gastrointestinal hemorrhage. Registry data tend to underreport “minor” hemorrhage due to several reasons, and the Extracorporeal Life Support Organization (ELSO), for example, in 2014 defined bleeding to be documented not before a blood loss of ≥20 ml/kg bw/24 h or ≥10 ml/kg bw/24 h RBC transfused (42). Prospective trials assessing bleeding draw a different picture: epistaxis and hematuria complicated treatment of ECMO patients in even two-thirds of all patients in a cohort of patients with veno-arterial and veno-venous ECMO (54). Almost every third vvECMO patient suffered from relevant bleeding, and bleeding was associated with poor survival (54). While bleeding from cannula insertion sites and mucous membranes hints to impairment of primary hemostasis and can be controlled by local interventions, 4 to 19% of patients develop spontaneous intracranial bleeding with a survival rate of only 20% (55–60). In the aforementioned data, also patients with veno-arterial ECMO are partly included. In vvECMO according to ELSO registry data, intracranial bleeding seems to be more frequent compared to vaECMO (52). The exact incidence of intracranial bleeding is difficult to assess, as not all ECMO patients routinely receive cranial imaging and not all of the deceased undergo cerebral autopsy.

## Transfusion

Continuous blood loss and hemolysis on ECMO also necessitate the transfusion of blood products. Transfusion thresholds, however, are mainly based on experts' opinions and differ between guidelines and recommendations. It is widely accepted that hemoglobin levels below 7–9 g/l should not be tolerated in adult vvECMO resulting in almost 90% of all patients receiving at least one packed red blood cell transfusion (RBC) during vvECMO (44, 52, 61). Treating anemia can improve oxygen delivery and may support coagulation at bleeding sites. Several studies, however, demonstrated that a restrictive threshold has acceptable outcomes in single-center cohorts (62). The recommended threshold to substitute platelet concentrates ranges from 50 to 100 thousand platelets/μl (44). Platelets are indispensable for primary hemostasis, but on the contrary platelet count does not necessarily correlate with platelet function; therefore, even with a platelet count above the aforementioned threshold, platelet transfusion may be reasonable in bleeding patients.

On the contrary, not indicated transfusions may be disadvantageous for patients: transfusions induce inflammatory cascades, have immunomodulatory effects, and are associated with the transmission or development of infections (63–66).

## Steps to Control Bleeding

### The “Treat Before They Bleed” Approach

**Figure 1:**
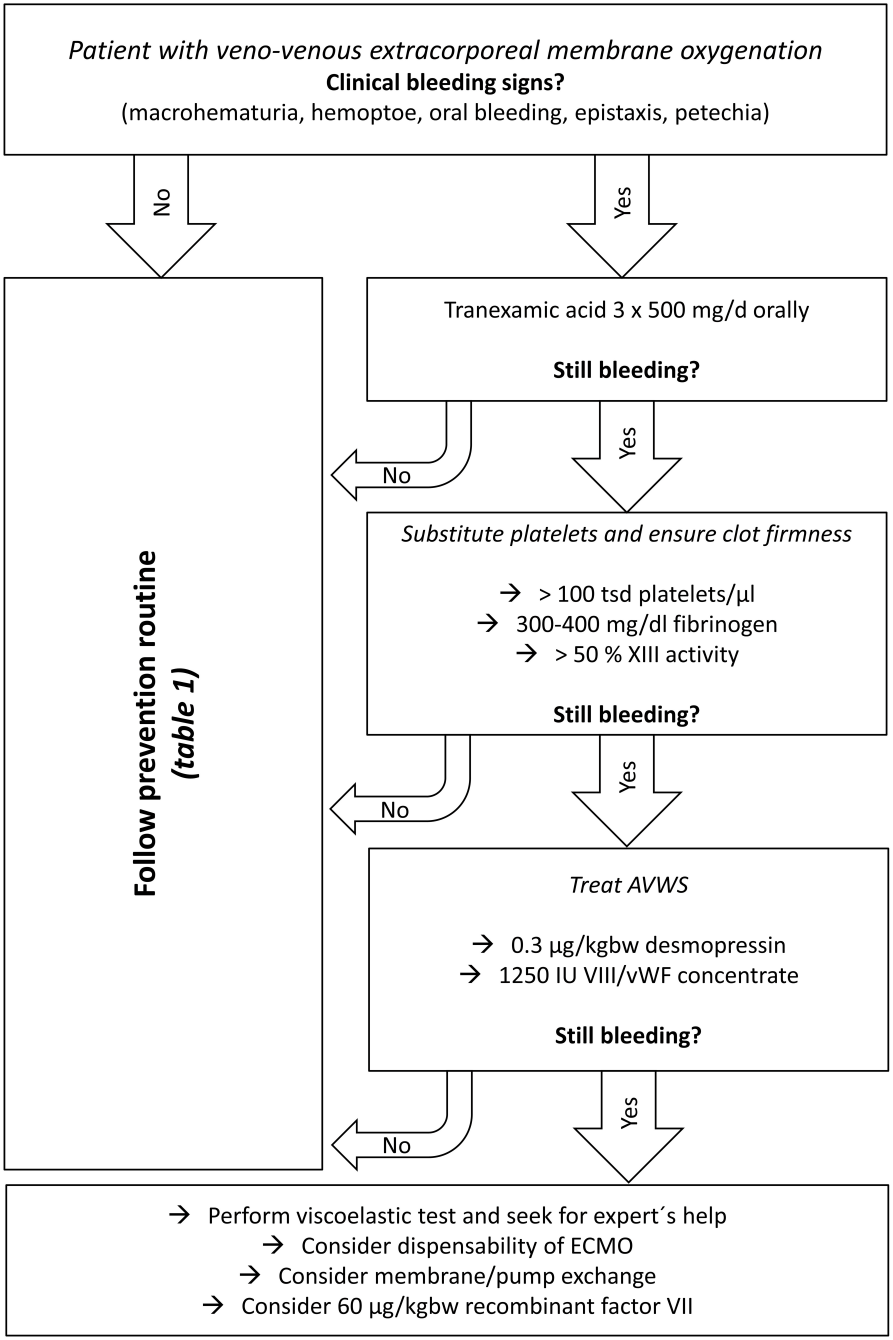
Departmental treatment algorithm for clinical bleeding in venovenous ECMO patients.

As spontaneous intracranial hemorrhage—the most feared complication—should be avoided and blood transfusions should be restricted, early diagnosis, and treatment of acquired bleeding disorders should be performed before clinical bleeding can be observed. In our center, we established a diagnosis and treatment protocol for vvECMO patients (Table 1). This is mainly based on the key points such as prevention of bleeding, minimized anticoagulation, and correction of acquired coagulation disorders. The protocol and the treatment algorithm (Figure 1) at the author's department go far beyond what is recommended in current international guidelines (44). This is due to the high experience of this center and made possible by the good availability of a coagulation laboratory.

**Table 1:** Routine blood drawings for the determination and treatment of acquired bleeding disorders during vvECMO in our department.

**Interval**	**Parameter**	**Target**	**Intervention**
Hourly	Hemoglobin	>10 g/dl	Red cell concentrate
Daily	Platelets (citrate) INR aPTT	>100 000/μl <1.35 <40 s	Platelet concentrate PPSB Fresh frozen plasma
Monday + Thursday and if clinical bleeding signs are present	Factor VIII-activity	>70 %	10 IU/kg Factor VIII concentrate i.v.
	Factor XIII-activity	>50%	1,250 IU Factor XIII concentrate i.v.
	vWF:Ag vWF:A	vWF:A/vWF:Ag ratio >0.73	0.3 μg/kg Desmopressin i.v. if target value not reached repetition of 0.2 μg/kg desmopressin i.v. if target value still not reached administration of vWF-containing concentrate (10 IU/kg i.v.)

*PPSB, Prothrombin complex concentrate; vWF:A, von Willebrand factor activity; vWF:Ag, von Willebrand factor antigen*.

### Minimizing Anticoagulation

Bleeding diathesis may be enhanced by anticoagulants (56, 67). Annich pointed out the “precarious balance of hemostasis during ECMO therapy” (48), and Ranucci described anticoagulation in extracorporeal therapy as “navigating difficult seas between thrombosis and bleeding” (68).

Reducing anticoagulation in vvECMO to prophylactic doses is safe and feasible and reduces the risk of bleeding. Krueger et al. reported data of more than 60 patients on vvECMO who received enoxaparin in only prophylactic dose and did not develop thromboembolic events or bleeding episodes. In combination with correction of acquired coagulation disorders, there was no increase in thrombotic pump failure (49). Incidence of bleeding and need for blood transfusion with a mean of only 1,15 RBC units and 0,6 fresh frozen plasma units per ECMO day was relevantly lower compared to other reported cohorts (35, 69, 70). Meanwhile, concepts with low dose or even without anticoagulation have only been applied in a few centers (71, 72). Low-molecular-weight heparin seems to be the better anticoagulant compared to UFH with regard to the prevention of thromboembolic events (50).

### Balancing Benefit and Risk With the Use of Anticoagulation and Transfusion in vvECMO

When patients begin to present with ongoing bleeding and transfusion of blood components becomes necessary during vvECMO, it should be critically revised whether the extracorporeal therapy still is beneficial for the patient. Probably, explantation of the vvECMO in favor of accepting a more invasive mechanical ventilation may be the less complicative approach.

### Control of Acquired Disorders of Primary Hemostasis

When preconditions like INR, aPTT, blood pH, temperature, and calcium levels are optimized and patients clinically still bleed, transfusion of platelet concentrates is a reasonable approach. Depending on the medical center, a particular platelet count has to be determined as trigger for transfusion. In our center, for example, we chose a transfusion threshold of >100 000 /μl platelets analogous to national guidelines for the treatment of traumatic brain injury (73). If bleeding still occurs after platelet transfusion, it should be taken in account that the bleeding symptoms may be due to impaired platelet function. Objectivation of platelet function in clinical practice is difficult, as point-of-care systems may fail to detect ECMO-induced platelet dysfunction. Therefore, calculated transfusion of platelets may be effective to control bleeding even when platelet count is still >100 000 platelets/μl.

Adhesion of platelets to collagen presented in case of endothelial injury is promoted by the von Willebrand factor. As described above, vvECMO leads to acquired impairment of vWF function, so-called AVWS. For detection of AVWS, at least determination of vWF: Ag and vWF: A is necessary (29). In case of a reduced vWF: A/vWF: Ag ratio, AVWS is likely. First-line treatment is therapy with desmopressin (DDVAP) with 0,3 μg/kgbw to induce secretion of vWF multimers stored in endothelial cells (28, 74, 75). The next step is the substitution of vWF-containing concentrates. The most common drug is factor VIII/vWF concentrate derived from human plasma (76). A new option is recombinant von Willebrand factor concentrate (77).

### Waste Coagulopathy

Diffuse activation of the coagulation system by platelet-derived microparticles, contact activation *via* factor XII, endothelial damage, and inflammation lead to waste coagulopathy (69, 78). In this context, deficiency of fibrinogen and/or factor XIII are common findings and may enhance bleeding diathesis (23, 35, 79–84). As fibrinogen is essential as well for clot formation as also for the activation of platelets, it is necessary to maintain normal fibrinogen levels by either transfusion of fresh frozen plasma (FFP) or fibrinogen concentrate. Clot firmness is dependent on cross-linking of fibrin with coagulation factor XIII (23, 79, 84–87). As many vvECMO patients present with factor XIII deficiency (23) which may enhance bleeding, substitution with factor XIII concentrates can be necessary to maintain clot firmness. In addition, hyper fibrinolysis should be diagnosed by viscoelastic tests like thrombelastography (88) and treated with infusion of tranexamic acid (89). Tranexamic acid may also be applied locally at bleeding sites.

### Exchange of Components

As bio-coating of the extracorporeal circuit continuously is washed away and coagulation activating fragments accumulate in the oxygenator membrane, an important step to control ongoing bleeding and hemolysis during prolonged vvECMO is complete exchange of extracorporeal pump and/or oxygenator membrane (90, 91). Cannulas may be left *in situ* and connected to the new system in order to minimize the risk of cannulation.

### Recombinant Factor VII

In case of uncontrollable bleeding during extracorporeal therapy despite optimization of the abovementioned preconditions, the careful use of recombinant factor VII has been described (92–94). This therapy has to be weighed carefully against the risk of acute clot formation within the extracorporeal system and possible thrombotic pump failure. Doses up to 60 μg/kgbw of recombinant factor VII seem to present an acceptable risk profile as ultima ratio option when all formerly described interventions have failed to control bleeding.

### Hypercoagulopathy Following ECMO

Only hours after explantation of vvECMO, vWF: A, vWF: CB, and factor VIII activity not only recover, but also become overcorrected beyond values before vvECMO. These findings imply that the risk of thromboembolic events is particularly high after vvECMO decannulation which needs to be taken into account when planning appropriate anticoagulation (21, 78). According to a standardized protocol in our center, all patients receive a subcutaneous dose of 80 IU/kg bw enoxaparin 1 h after decannulation. According to the anti-Xa parameter (4 h later), the following doses of enoxaparin are adjusted aiming for an aXa of 0.5–0.8 IU/ml (therapeutic anticoagulation). Therapeutic anticoagulation with enoxaparin or rivaroxaban is maintained for at least 6 weeks (even if the patient is meanwhile discharged from the hospital).

## Summary

- Treat before they bleed
- Decrease anticoagulation
- Control primary hemostasis
- Substitute wasted coagulation factors
- Consider membrane exchange and ECMO explantation
- Do not underestimate hypercoagulatory status after ECMO explantation.

## Author Contributions

JK and BZ did the research of relevant part of articles cited. They performed literature search. JK wrote the primary version of the manuscript and drew the pictures and tables. BZ revised the manuscript. Both authors agreed to the final version of the manuscript. Both authors contributed to the article and approved the submitted version.

## Funding

The article processing charge was partly funded by the Baden-Wuerttemberg Ministry of Science, Research and Art and the University of Freiburg in the funding programme Open Access Publishing.

## Conflict of Interest

The authors declare that the research was conducted in the absence of any commercial or financial relationships that could be construed as a potential conflict of interest.

## Publisher's Note

All claims expressed in this article are solely those of the authors and do not necessarily represent those of their affiliated organizations, or those of the publisher, the editors and the reviewers. Any product that may be evaluated in this article, or claim that may be made by its manufacturer, is not guaranteed or endorsed by the publisher.
